# Post-radiotherapy maintenance treatment with fluticasone propionate and salmeterol for lung cancer patients with grade III radiation pneumonitis: A case report

**DOI:** 10.1097/MD.0000000000010681

**Published:** 2018-05-25

**Authors:** Pingping Zhang, Hongxia Yan, Sheng Wang, Jindan Kai, Guoliang Pi, Yi Peng, Xiyou Liu, Junwei Sun

**Affiliations:** a Department of Radiation Oncology; b Department of Surgical Oncology, Hubei Cancer Hospital, the Affiliated Hubei cancer Hospital of Huazhong University of Science and Technology, Wuhan, China.

**Keywords:** fluticasone propionate, inhaled seretide, non-small-cell lung cancer, radiation pneumonitis, relapse, salmeterol

## Abstract

**Rationale:**

This combination of fluticasone propionate (FP) and the long-acting β2-agonist salmeterol (Salm) can control the symptoms of asthma and COPD better than FP or Salm on their own and better than the combination of inhaled corticosteroids plus montelukast. FP/Salm has been shown to control symptoms of asthma and COPD better than a double dose of inhaled steroids. The patient in our report had a history of COPD, and suffered relapse of RP when given only steroids. It is possible that COPD history helps explain this patient's more difficult treatment course. Therefore, this combination may be more effective than inhaled steroids for patients with a history of COPD.

**Patient concers:**

This patient suffered adverse reactions triggered by methylprednisolone: weight gain, hyperglycaemia and sleep disturbance after more than two months of intravenous and oral prednisolone. These reactions disappeared when we switched the patients to FP/Salm maintenance therapy.

**Diagnoses:**

The patient underwent upper right lobectomy in September 2011. Immunohistochemistry indicated low squamous cell differentiation, and he was diagnosed with stage IIB disease (T2N1M0) according to the Union for International Cancer Control (UICC) (7th edition).

One month after repeat radiotherapy, the patient experienced fever (37.6°C), cough, chest distress and shortness of breath. We performed serologic tests, laboratory tests for procalcitonin and C-reactive protein, as well as sputum and blood cultures to rule out bacterial infection. Chest CT showed consolidation with air bronchogram in the hilum of the right lung and ground-glass densities in the right lower lobe and left upper lobe. These radiographic signs are typical of RP. Since the patient required oxygen, he was diagnosed with grade III RP.

**Interventions:**

After the patinet was diagnosed with grade III RP. The patient was immediately prescribed oxygen, anti-infectives for prophylaxis, treatments to facilitate expectoration and prevent asthma, and most importantly, intravenous methylprednisone at an initial dose of 60  per day. And we cut the steroid dose in half every one week when the patient's symptoms improved obviously, and the patchy shadow on the chest radiograph sharply reduced. Then we give him FP (500 mg)/Salm (50 mg) twice daily for two months. Then the dose was halved for an additional two months.

**Outcomes:**

The patient showed no signs of tumor or RP relapse by the last follow-up in March 2018.

**Lessons:**

This maintenance therapy of FP/Salm for patient with grade III RP may help avoid relapse when steroid therapy is tapered, particularly for patients with a history of COPD. It may also reduce risk of steroid-associated adverse effects. Based on the results observed with our patient, we intend to design a prospective trial to assess the efficacy of FP/Salm when used as preventive treatment for patients at high risk of RP, and when used as maintenance treatment for patients with grade III RP.

## 1 Introduction

Radiotherapy plays a major role in the radical treatment of patients with non-small cell lung cancer.^[1]^ Radiation pneumonitis (RP) is a fairly common subacute side effect of radiotherapy in lung cancer patients, with a reported incidence of 10% to 30%. This broad incidence range likely reflects differences among patient subpopulations, the subjectivity of scoring RP, and treatment-related factors.^[2]^ RP develops in the first few weeks or months after radiotherapy with symptoms of dyspnea, nonproductive cough, pleuritic chest pain, fever, and, in rare cases, acute respiratory distress syndrome.^[3,4]^ RP can be reversible, but in severe cases, it may be life-threatening. High-dose intravenous steroids are recommended for suppressing the symptoms of grade III RP, which is defined by a requirement for oxygen.^[5]^

Systemic steroids must be taken for approximately 2 months to prevent the exacerbation of symptoms,^[5,6]^ yet systemic steroid therapy lasting >1 month is associated with increased risk of systemic side effects such as weight gain, hyperglycaemia, sleep disturbances, mood changes, and oedema. Another problem with steroid therapy is that relapses occur in many patients after the dose is tapered.^[7]^

Inhaled steroids, which are associated with lower risk of systemic side effects than systemic steroids,^[8,9]^ can control symptoms of grade II RP. In one study, 18 of 24 patients showed a significant improvement in clinical symptoms, whereas the remaining 6 (25%) patients were nonresponders to inhaled steroids.^[10]^ Similarly, in the specific case of using inhaled corticosteroids to treat inflammation in patients with asthma or chronic obstructive pulmonary disease (COPD), many patients continue to experience troublesome symptoms, exacerbations, and compromised lung function.^[11]^ To address both the inflammatory and bronchoconstrictive components of asthma and COPD, fluticasone propionate (FP) and the long-acting β2-agonist salmeterol (Salm) were combined into a single formulation.^[10]^ This combination can control the symptoms of asthma and COPD better than FP or Salm on their own^[12,13]^ and better than the combination of inhaled corticosteroids plus montelukast.^[11]^

Here we describe 1 patient who experienced grade III RP after radiotherapy for lung tumors, and who was treated with intravenous steroids for 2 to 3 weeks, followed by FP/Salm for 4 months. This long-term maintenance therapy was able to avoid symptom exacerbation, steroid-related adverse reactions, and relapse.

## 2 Case report

### 2.1 Patient undergoing repeat radiotherapy

**Figure 1:**
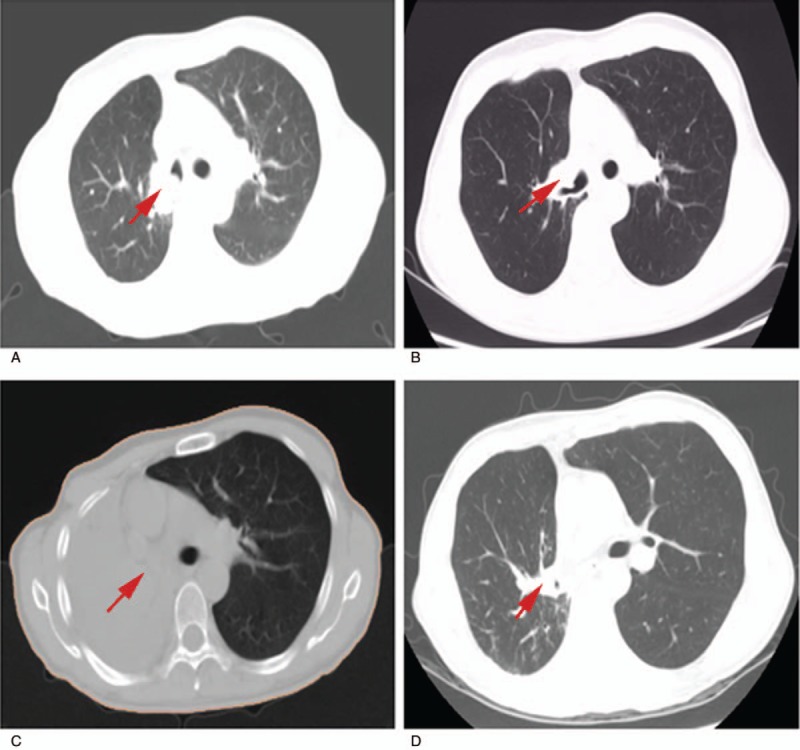
Chest computed tomography of the lung tumor patient undergoing repeat radiotherapy. (A) Recurrence of disease in the hilum of the right lung after surgery. (B) Partial response at the end of the first radiotherapy. (C) Recurrent disease again in the right lung. (D) After repeat radiotherapy, the enlarged tumor in the right lung shrank significantly and the atelectasis nearly disappeared.

**Figure 2:**
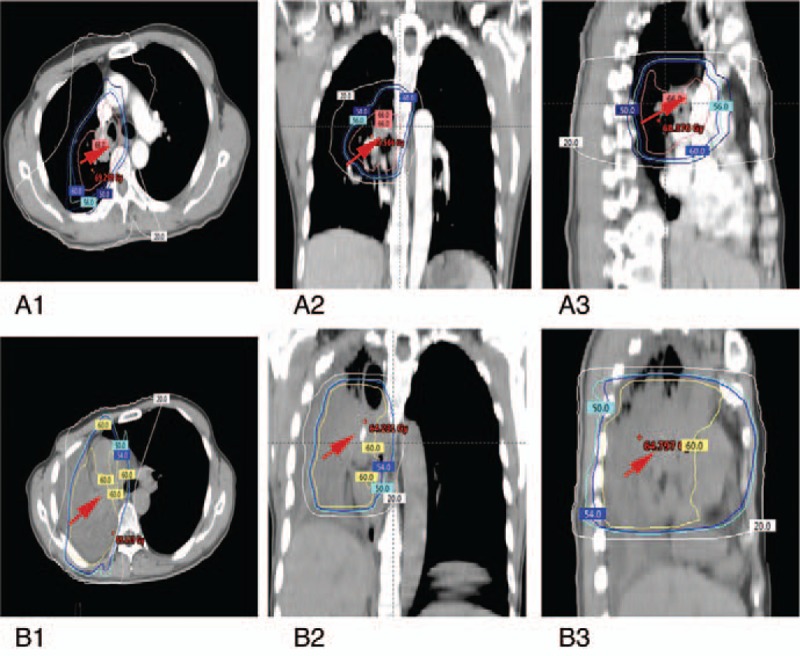
(A, B) Dosimetry of the first radiotherapy (A1–3) and repeat radiotherapy (B1–3) of the patient.

A lung tumor was accidentally discovered in a 54-year-old Chinese man with a 20-year history of smoking when he underwent computed tomography (CT). The patient underwent upper right lobectomy in September 2011. Immunohistochemistry indicated low squamous cell differentiation, and he was diagnosed with stage IIB disease (T2N1M0) according to the Union for International Cancer Control (7th edition). He received 4 cycles of chemotherapy with gemcitabine and cisplatin, and subsequently he was followed up every 3 months. In March 2013, follow-up CT revealed recurrent disease in the hilum of the right lung (Fig. 1A). He received 2 cycles of salvage chemotherapy with docetaxel and cisplatin, but follow-up CT showed disease progression. The patient received intensity-modulated radiotherapy (IMRT) at a dose of 64 Gy in 32 fractions at the locoregionally recurrent lesion (Fig. 2A1–3). Partial response was observed by the end of radiotherapy based on CT (Fig. 1B).

In March 2015, the patient presented with chest distress and shortness of breath, malaise, fatigue, cough, and an enlarged tumor in the hilum of the right lung and atelectasis of almost the right lung based on CT (Fig. 1C). The patient refused chemotherapy and so was treated instead with salvage IMRT for local failure at a dose of 60 Gy in 30 fractions (Fig. 2B1–3) with the following dosimetry: left lung V5, 21%; left lung V20, 6%; maximum heart dose, 61.1 Gy; V40, 30%; V30, 39%; and maximum spinal cord dose in the dose overlap region, 23.2 Gy. The patient's respiratory symptoms improved noticeably during radiotherapy. CT revealed that the enlarged tumor in the right lung had shrunk significantly, and that the atelectasis had nearly disappeared (Fig. 1D).

**Figure 3:**
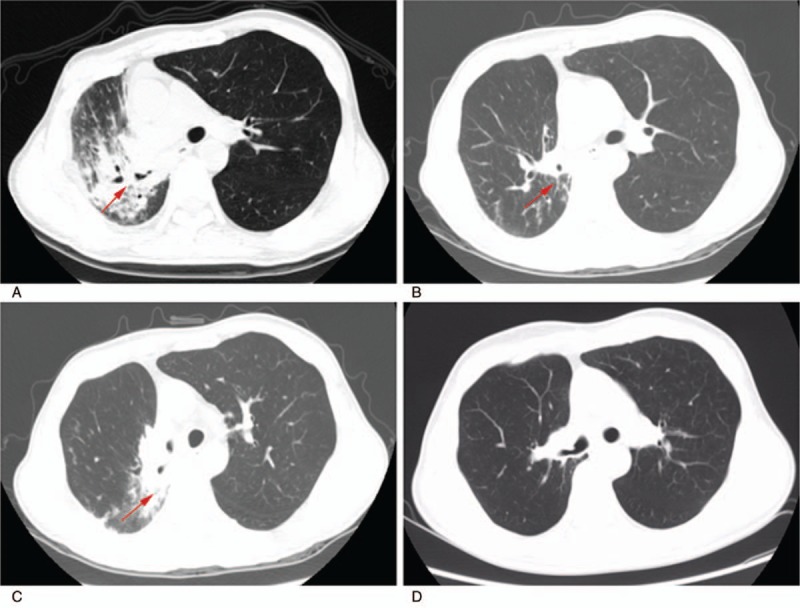
Chest computed tomography of the lung tumor patient after repeat radiotherapy. (A) At 1 month after repeat radiotherapy, consolidation with air bronchogram in the hilum of the right lung and ground-glass densities in the right lobe were observed. (B) After intravenous methylprednisone, the patchy shadow on the chest radiograph nearly disappeared. (C) At 1 month after tapering oral prednisolone, a consolidated shadow was observed again inside the irradiated volume. (D) At 24 months after FP/Salm maintenance therapy, complete resolution was observed in the right lung.

One month after this repeat radiotherapy, the patient experienced fever (37.6°C), cough, chest distress, and shortness of breath. The patient's laboratory results at that time are shown in Table 1. We performed serologic tests, laboratory tests for procalcitonin, and C-reactive protein, as well as sputum and blood cultures to rule out bacterial infection. Chest CT showed consolidation with air bronchogram in the hilum of the right lung and ground-glass densities in the right lower lobe and left upper lobe (Fig. 3A). These radiographic signs are typical of RP. As the patient required oxygen, he was diagnosed with grade III RP. The patient was immediately prescribed oxygen, anti-infectives for prophylaxis, treatments to facilitate expectoration andprevent asthma, and most importantly, intravenous methylprednisone at an initial dose of 60 mg/day. After 1 week, the patient's respiratory symptoms improved obviously, and the patchy shadow on the chest radiograph disappeared almostly (Fig. 3B). At this point, we cut the steroid dose in half. After another week, the steroid dose was halved again. Then the patient was switched to an equivalent dose of oral prednisolone, which was tapered and discontinued after 8 weeks. During systemic prednisolone therapy, the patient experienced systemic side effects of weight gain, hyperglycaemia, and sleep disturbance.

**Table 1:**
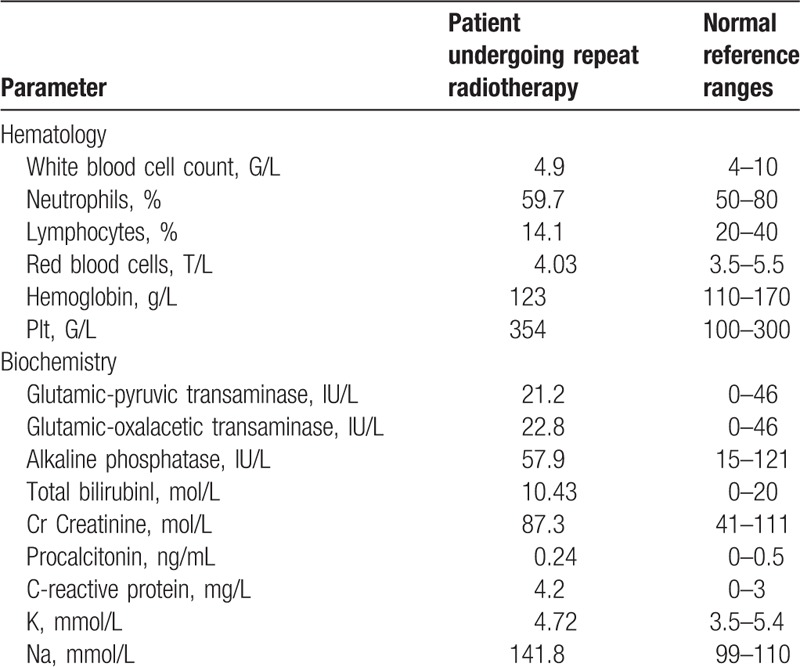
Patient laboratory results.

One month after the end of oral prednisolone therapy, the patient again developed a productive cough with a low fever, chest distress, and shortness of breath. Chest CT again showed a consolidated shadow inside the irradiated volume (Fig. 3C), and the patient was diagnosed with recurrent RP. We administered intravenous methylprednisolone at an initial dose of 60 mg/day for 1 week, then we halved the dose for another week. The patient's symptoms improved obviously, and the patchy shadow on the chest radiograph nearly disappeared. As the patient had a history of COPD associated with chronic bronchitis, which was brought under control in 2006 using Seretide (FP 500 μg and Salm 50 μg), and as the patient had reacted adversely to previous systemic steroid therapy, we decided to give him FP (500 mg)/Salm (50 mg) twice daily for 2 months. Then the dose was halved for an additional 2 months. The patient showed no signs of tumor or RP relapse by the last follow-up in March 2018 (Fig. 3D).

## 3 Discussion

RP is one of the most frequent dose-limiting toxicities arising from thoracic radiotherapy, especially in lung cancer patients who are older and frequently have reduced lung function caused by tumor's mass effect or preexisting lung disease.^[14]^ The patient in the present report had reduced lung function caused by large, centrally located recurrences and atelectasis of almost the right lung. He suffered from grade III RP 1 month after radiotherapy, which manifested as symptoms of cough, shortness of breath, and low-grade fever.

High-dose intravenous methylprednisolone for 1 week led to marked improvement of symptoms and obvious shrinkage of the patchy shadow on the chest radiograph. However, this patient suffered adverse reactions triggered by methylprednisolone: weight gain, hyperglycaemia, and sleep disturbance after >2 months of intravenous and oral prednisolone. These reactions disappeared when we switched the patients to FP/Salm maintenance therapy. An alternative approach could be to deliver inhaled steroids, which appear to trigger fewer side effects than systemic steroids,^[8,9]^ including when treating RP.^[15]^ One recent study has suggested that symptomatic patients with grade II RP can be initially treated with inhaled steroids rather than systemic steroids.^[16]^ However, this approach would probably not have been as effective as FP/Salm for our case because he had grade III pneumonitis with cough, shortness of breath, and fever and therefore required immediate attention; intravenous application of steroids was crucial to control inflammation as soon as possible. Moreover, this patient was suffering from a relapse of grade III RP. Such relapse of respiratory symptoms has been observed in many patients following systemic steroid therapy. Some reports describe patients who required corticosteroid therapy for >3 years^[17]^ or who were refractory to corticosteroids.^[18]^

The patient in the present report took FP/Salm maintenance therapy for 4 months. Although this may appear to be a long time, oral steroids should be taken for at least 2 months to avoid symptom exacerbation in grade II RP^[6]^ (see also the DEGRO S2 guidelines [version 1.2] released in February 2015). We tapered the FP/Salm dose only if chest CT at 8 weeks after the start of maintenance therapy showed regression of the typical radiographic signs of RP. The efficacy of this strategy is suggested by the fact that our patient did not experience symptom exacerbation during the stepwise dose reduction of FP/Salm.

The patient in our report had a history of COPD, and suffered relapse of RP when given only steroids. It is possible that COPD history helps explain this patient's more difficult treatment course, but associations between COPD and RP are unclear. Some studies suggest that COPD significantly increases risk of RP,^[19,20]^ whereas other studies suggest it does not.^[21–23]^ This discrepancy may relate to the patient's lung function level.^[14]^

Just as links between COPD and risk of RP are controversial, so are links between COPD and responsiveness to steroid therapy. One study suggested that an association between COPD and partial response may explain the non-response of several patients to inhaled steroids, given that the patients’ clinical symptoms, such as dyspnea, were also typical of COPD exacerbation.^[16]^ Whether a link between COPD and steroid responsiveness exists or not, FP/Salm has been shown to control symptoms of asthma and COPD better than a double dose of inhaled steroids.^[11]^ Therefore, this combination may be more effective than inhaled steroids for patients with a history of COPD.

To prevent the occurrence of RP, the irradiated volume during radiotherapy should be as low as possible. There is considerable evidence that the risk of late lung toxicity is a function of dose-volume parameters such as the mean lung dose and cumulative doses received by certain lung volumes. As a general rule, the risk for symptomatic RP sharply increases with a mean lung dose >20 Gy, V20 >30%, and V30 >20%.^[24,25]^ For our patient, mean lung dose was kept <15 Gy, mean V20 <27%, and V30 <20%.

Smoking may be a protective factor against developing RP,^[26]^ and it may be associated with better response to inhaled steroids. This may be because of differences in immune responses between smokers and nonsmokers.^[27]^ Our patient had decades-long smoking histories and benefited from FP/Salm as maintenance treatment. Therefore, this treatment may be particularly effective for patients with grade III RP and a long smoking history.

Our patient suffered from grade III RP after intensity-modulated radiotherapy at a dose of 60 Gy in 30 fractions. Although this type of radiotherapy is a standard option for treating recurrent non-small cell lung cancer and is associated with acceptable toxicity, stereotactic body radiation therapy may offer lower toxicity.^[28,29]^ Unfortunately, this type of radiotherapy was not suitable for our patient because of a recurrent tumor in the hilum of the right lung and atelectasis involving almost the right lung.

Success of FP/Salm maintenance therapy requires that clinicians have considerable experience with RP and that they observe the patients closely.

## 4 Conclusions

This is the first report on the efficacy of FP/Salm as maintenance therapy for patient with grade III RP in whom systemic steroid therapy has brought respiratory symptoms under control. This maintenance therapy may help avoid relapse when steroid therapy is tapered, particularly for patients with a history of COPD. It may also reduce risk of steroid-associated adverse effects. Further clinical trials in Asian countries are needed to assess the safety and efficacy of FP/Salm for grade III RP. Future studies should examine effective dosages, timing of therapy, and responsiveness of different patient populations. Based on the results observed with our patient, we intend to design a prospective trial to assess the efficacy of FP/Salm when used as preventive treatment for patients at high risk of RP, and when used as maintenance treatment for patients with grade III RP.

## Acknowledgments

The authors thank all our colleagues who helped us in the care of the patient.

## Author contributions

PPZ, HXY, SW and JK carried out the data collection and writing of the manuscript; PPZ helped to conceive the study; JWS contributed to the design of the study; GLP helped to collect data; YP and XYL analysed the radiological images. All authors read and approved the final manuscript.

**Data curation:** Pingping Zhang.

**Formal analysis:** Pingping Zhang, Hongxia Yan, Guoliang Pi.

**Funding acquisition:** Junwei Sun.

**Methodology:** Pingping Zhang, Yi Peng.

**Resources:** Sheng Wang, Jindan Kai.

**Supervision:** Junwei Sun.

**Visualization:** Junwei Sun.

**Writing – original draft:** Pingping Zhang.

**Writing – review & editing:** Pingping Zhang, Xiyou Liu.
